# Generation of a Convalescent Model of Virulent *Francisella tularensis* Infection for Assessment of Host Requirements for Survival of Tularemia

**DOI:** 10.1371/journal.pone.0033349

**Published:** 2012-03-12

**Authors:** Deborah D. Crane, Dana P. Scott, Catharine M. Bosio

**Affiliations:** 1 Immunity to Pulmonary Pathogens, Laboratory of Intracellular Parasites, Rocky Mountain Laboratories, NIAID, National Institutes of Health, Hamilton, Montana, United States of America; 2 Veterinary Pathology Section, Rocky Mountain Veterinary Branch, Rocky Mountain Laboratories, NIAID, National Institutes of Health, Hamilton, Montana, United States of America; University of Louisville, United States of America

## Abstract

*Francisella tularensis* is a facultative intracellular bacterium and the causative agent of tularemia. Development of novel vaccines and therapeutics for tularemia has been hampered by the lack of understanding of which immune components are required to survive infection. Defining these requirements for protection against virulent *F. tularensis*, such as strain SchuS4, has been difficult since experimentally infected animals typically die within 5 days after exposure to as few as 10 bacteria. Such a short mean time to death typically precludes development, and therefore assessment, of immune responses directed against virulent *F. tularensis*. To enable identification of the components of the immune system that are required for survival of virulent *F. tularensis*, we developed a convalescent model of tularemia in C57Bl/6 mice using low dose antibiotic therapy in which the host immune response is ultimately responsible for clearance of the bacterium. Using this model we demonstrate αβTCR^+^ cells, γδTCR^+^ cells, and B cells are necessary to survive primary SchuS4 infection. Analysis of mice deficient in specific soluble mediators shows that IL-12p40 and IL-12p35 are essential for survival of SchuS4 infection. We also show that IFN-γ is required for survival of SchuS4 infection since mice lacking IFN-γR succumb to disease during the course of antibiotic therapy. Finally, we found that both CD4^+^ and CD8^+^ cells are the primary producers of IFN-γand that γδTCR^+^ cells and NK cells make a minimal contribution toward production of this cytokine throughout infection. Together these data provide a novel model that identifies key cells and cytokines required for survival or exacerbation of infection with virulent *F. tularensis* and provides evidence that this model will be a useful tool for better understanding the dynamics of tularemia infection.

## Introduction

*Francisella tularensis* is a Gram negative, facultative intracellular pathogen. Currently, there are five major subspecies of *F. tularensis*. Little is known about subspecies *philomargia* and *medasiatica* with the exception that they are not considered pathogenic to the immunocompetent host [1], [2]. Subspecies *novicida* is also relatively non-pathogenic to humans [2]. However, it has been employed by many laboratories as a model intracellular pathogen that resides in the cytosol of host cells. Subspecies *holarctica* can cause disease in humans, but is rarely fatal. However, this subspecies readily infects and causes lethal disease in mice [3], [4]. Perhaps more importantly, further attenuation of subspecies *holarctica* resulted in generation of the Live Vaccine Strain (LVS) [5]. Although LVS is no longer routinely used in the United States as a vaccine against tularemia, this strain exhibits route and dose dependent virulence in mice. Furthermore, mice that survive sublethal challenge with LVS are immune to secondary lethal challenge [6]. Together, and similar to *F. novicida*, these phenomena make LVS infection in mice an interesting model for requirements of immunity against intracellular pathogens.

Subspecies *tularensis* was the first isolated organism of the *F. tularensis* species and, in stark contrast to other subspecies, is highly pathogenic for humans [7], [8]. Exposure to as few as 15 bacteria can result in severe, potentially lethal, disease. Mice are also highly susceptible to infection with subspecies *tularensis*, and serve as a useful rodent model to study this pathogen. However, as observed in humans, evidence of early immune responses directed against strains of *F. tularensis* ssp. *tularensis* (such as SchuS4) is not apparent [9], [10]. Further, when paired with a short mean time to death, e.g. 5 days, dissection of the requirements for immunity against subspecies *tularensis* has been difficult.

Despite variability in virulence, all tested subspecies of *F. tularensis* appear to target phagocytic cells for replication, readily escaping into the cytosol following receptor mediated uptake [11], [12]. Thus, one solution to the challenges posed by infection of mice with virulent *F. tularensis* was to use either attenuated *novicida* or *holarctica*, specifically LVS, as surrogates for infection with highly virulent strains. However, over the last several years it has become apparent that the interactions of attenuated and virulent subspecies of *F. tularensis* with host cells are not the same. For example, evasion of stimulation of rapid pro-inflammatory responses is considered a primary mechanism of virulence for subspecies *tularensis* [9], [13]–[16]. In contrast, both subspecies *novicida* and strain LVS induce inflammatory responses in human cells [17]–[19]. We recently reported that virulent *F. tularensis* induces IFN-β as a mechanism to suppress IL-12, whereas LVS infection failed to exhibit this activity [20]. Virulent *F. tularensis* also possesses the ability to utilize the host plasminogen system for degradation of opsonizing antibodies. LVS lacks the ability to engage the host plasminogen system for this purpose [21]. In vivo, *novicida* induces rapid recruitment of granulocytic cells, whereas recruitment of these cells following infection with subspecies *tularensis* is markedly delayed [15]. Thus, it is unclear if the mechanisms by which *novicida* or LVS manipulate the host immune system will be consistent with those utilized by fully virulent subspecies *tularensis*.

In addition to variability in the pathogenesis of infection with attenuated versus virulent strains, there are also inconsistent results obtained from different labs studying the same attenuated strains of *F. tularensis*. For example, early analysis of LVS infection in mice deficient for IL-12p40 and IL-12p35 suggested that neither of these cytokines were required to survive LVS infection [22]. However, later publications using the same genetically deficient mice presented data that both IL-12p35 and IL-12p40 were required for survival in LVS infection [23]. Similarly, it has been suggested that induction of IL-4 following LVS infection of macrophages exacerbates disease [24]. In contrast, other laboratories have shown that production of IL-4 aids in clearance of LVS infection and that LVS can inhibit inflammation mediated, in part, by IL-4 [25], [26]. Furthermore, the ability of attenuated strains to provoke cytokine production and activate the inflammasome among infected host cells has also been a point of controversy [27]–[30]. Given the contrasting results obtained with attenuated subspecies, and the difference in pathogenesis between virulent and attenuated subspecies, it is difficult to apply findings from infection with attenuated subspecies to those mediated by fully virulent *F. tularensis*. Together this emphasizes the importance for assessment of the requirements of protection against tularemia using fully virulent strains of *F. tularensis*.

A small number of studies with virulent *F. tularensis* have successfully identified requirements for survival of infection in vaccinated mice and host components that exacerbate disease in naive animals [31]–[35]. However, most experiments designed to determine host requirements for immunity against virulent *F. tularensis* in vivo are hampered by the low dose of bacteria capable of causing uniformly lethal disease and a very short mean time to death, i.e. 10–15 bacteria and approximately 5 days, respectively. Development of a model using virulent *F. tularensis* that allows for examination of bacterial-host interactions over longer periods of time would potentially resolve this problem for studying immunity to tularemia.

As a bacterium, *F. tularensis* can be killed by antibiotics. Therefore, antibiotics could be used to control and/or clear infection with virulent *F. tularensis* in experimentally infected animals. This process may extend the course of primary infection and allow investigation of the role of specific host cells and soluble mediators that play a role in tularemia. Previous studies have shown that treatment with the fluoroquinolone levofloxacin in Balb/c mice aids in clearance of SchuS4 following intranasal infection [36]. Furthermore, antibiotic treated Balb/c mice were resistant to secondary challenge with SchuS4, a feature that was attributed to the production of antibodies directed against Francisella. However, the requirement for other immune components was not investigated. These studies would be difficult in Balb/c mice for the practical reason that the majority of mice with specific genetic deficiencies, which would allow assessment of the role of specific cells and molecules, are bred on a C57Bl/6 background and not Balb/c.

The goal of the study presented herein was to develop a model of convalescent tularemia in C57BL/6 in which the host immune response, rather than antibiotic alone, ultimately controlled infection. This model would then be used to assess the requirement for specific cells and soluble mediators for control and/or resolution of disease mediated by virulent *F. tularensis*. Additionally, this model would result in median survival, e.g. 50–60%, of wild type animals to enable assessment of host components that either aid in resolution or exacerbation of disease. Herein, we describe the generation of such a model in C57BL/6 mice and identify key host cells and cytokines required to survive primary SchuS4 infection.

## Results

### Survival of *F. tularensis* SchuS4 infection following delivery of low dose antibiotic

**Figure 1:**
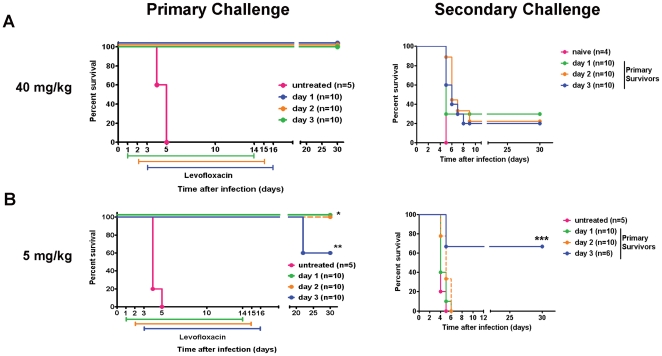
Dose and timing of antibiotic therapy following intranasal infection with virulent *F. tularensis* ssp *tularensis* strain SchuS4. Groups of 5–10 C57Bl/6 mice were intranasally infected with 50 CFU SchuS4 in 25 µl. At the indicated time points after infection mice were injected intraperitoneally once daily with 40 (A) or 5 mg/kg (B) LVF diluted in 5% dextrose water. All mice were treated for 14 days. Thirty days after primary challenge all surviving animals (primary survivors) were re-challenged (secondary challenge) intranasally with 50 CFU SchuS4. * = p<0.05 compared to untreated controls and mice receiving antibiotic from day 3–16. ** = p<0.05 compared to untreated controls and mice receiving LVF on day 3. *** = p<0.05 compared untreated mice. Data in each graph is representative of 4 experiments of similar design.

Antibiotics can be used in humans and mice to effectively treat tularemia [37]–[39]. A recent report demonstrated intraperitoneal injection of 40 mg/kg of the fluoroquinolone levofloxacin (LVF) delivered daily for 14 days aids in the resolution of virulent *F. tularensis* infection in Balb/c mice [36]. However, efficacy of LVF in C57Bl/6 mice has not been examined. Since the majority of mice with targeted deletions in genes participating in immune responses are on a C57Bl/6 background, we first tested a dose of 40 mg/kg of LVF for its ability to enhance survival of intranasal SchuS4 infection in C57Bl/6 mice. Similar to reports in Balb/c mice, 100% of C57Bl/6 mice receiving 40 mg/kg of LVF beginning on day 1, 2 or 3 after infection with SchuS4 survived (Figure 1A). However, in contrast to data obtained from Balb/c mice, surviving C57Bl/6 mice are poorly protected against a second challenge of SchuS4 (Figure 1A). Specifically, 30% of animals treated with 40 mg/kg LVF beginning on day 1 of infection survive secondary challenge, whereas only 20% of animals receiving this dose of LVF on day 2 or 3 of primary infection survive secondary challenge (Figure 1A).

Survival of approximately 50–60% of animals treated with LVF would enable in vivo assessment of host components that contribute to either the resolution or exacerbation of primary SchuS4 infection. Treatment of mice with 40 mg/kg LVF did not result in survival rates that fell within this range. Therefore, we next tested a lower dose of LVF for its ability to aid in resolution of SchuS4 infection with the goal of reducing survivorship from 100% to approximately 50%. Similar to mice that received 40 mg/kg on day 1 or 2 after infection, all mice receiving 5 mg/kg LVF beginning on day 1 or 2 after intranasal infection survive (Figure 1B). However, unlike the minimal survival of secondary infection observed in mice treated with 40 mg/kg LVF, none of the mice given 5 mg/kg beginning on day 1 or 2 after infection survive secondary challenge with SchuS4 (Figure 1B). In contrast, delay of initiation of LVF therapy to day 3 of primary SchuS4 infection resulted in survival of 60% of the mice (Figure 1B). All mice that received LVF treatment beginning on day 3 of infection and survived to 30 days post-infection had circulating antibodies directed against *F. tularensis*, indicating all mice had been infected with SchuS4 (Figure S1). Furthermore, animals treated with 5 mg/kg beginning on day 3 of primary SchuS4 infection also exhibited the best survival of secondary challenge with approximately 66% of mice surviving a second intranasal infection with SchuS4. Thus, delivery of a low dose LVF (i.e. 5 mg/kg) beginning on day 3 of primary intranasal SchuS4 infection results in a percentage of survival following primary and secondary challenge that will allow assessment of host components required for resolution or exacerbation of both primary and secondary intranasal SchuS4 infection.

### Kinetics of SchuS4 replication following delivery of low dose LVF

**Figure 2:**
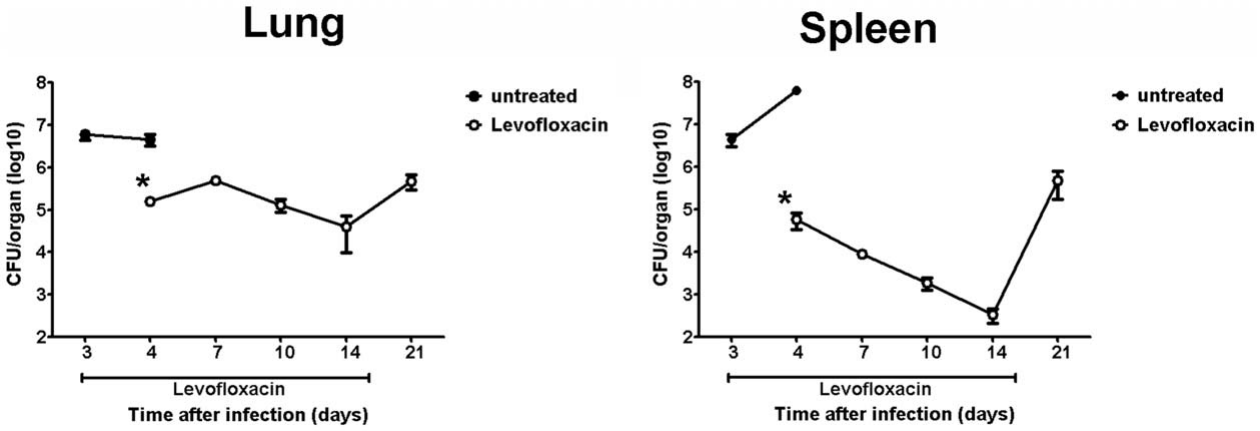
Clearance of SchuS4 following antibiotic therapy. Mice were infected intranasally with 50 CFU SchuS4. Three days after infection mice were treated once daily with 5 mg/kg LVF diluted in 5% dextrose water intraperitoneally for 14 days. At the indicated time points, animals (n = 5/group) were euthanized and lungs and spleens were assessed for bacterial loads. Error bars represent SEM. * = p<0.05 compared to untreated controls. Data is representative of 2 experiments of similar design.

We next determined if survival of SchuS4 infection following antibiotic therapy correlated with control of bacterial replication in target organs. Our goal was to establish a persistent infection that was cleared only after antibiotic therapy was stopped. Mice were infected with SchuS4 and treated with 5 mg/kg LVF as described in the Materials and Methods. At the indicated time points, lungs and spleens were assessed for bacterial loads. Within 24 hours of treatment, LVF restricted splenic and pulmonary replication of SchuS4 (Figure 2). Throughout the course of LVF therapy, bacterial numbers in the spleen continued to decrease to nearly undetectable numbers 14 days after infection (11 days of LVF treatment) (Figure 2). However, within 5 days of stopping LVF treatment, numbers of SchuS4 recovered from the spleen were similar to those observed in LVF treated animals 4 days after infection (1 day after LVF treatment) (Figure 2). In contrast to the reduction of SchuS4 in the spleen, LVF treatment had a smaller impact on the number of bacteria present in the lung. We observed approximately 1 log10 reduction of SchuS4 in the lungs of LVF treated animals during LVF therapy as opposed to nearly 2 log10 reduction in the spleen (Figure 2). Furthermore, after LVF treatment ended, we observed a modest increase in the numbers of SchuS4 in the lung (Figure 2). Surprisingly, despite the increase in bacterial numbers in the lung and spleen 21 days after infection, no bacteria were detected in animals that survived to 30 days after infection (data not shown). Infection of these survivors was confirmed by testing sera for antibodies directed against Francisella. Thirty days after infection, all surviving animals had measurable titers of circulating anti-Francisella antibodies (Figure S1). Thus, survival of SchuS4 infection following treatment with antibiotic correlates with control of bacterial replication during LVF therapy and eventual clearance of the organisms within 2 weeks after the course of antibiotic therapy is complete. Furthermore, these data suggest that treatment with low doses of antibiotic alone is not sufficient for clearance of SchuS4. Rather, eradication of the bacterium is dependent on appropriate host immune response directed against this pathogen which is present when antibiotic is withdrawn.

### Pathological Changes following SchuS4 infection and low dose LVF therapy

**Figure 3:**
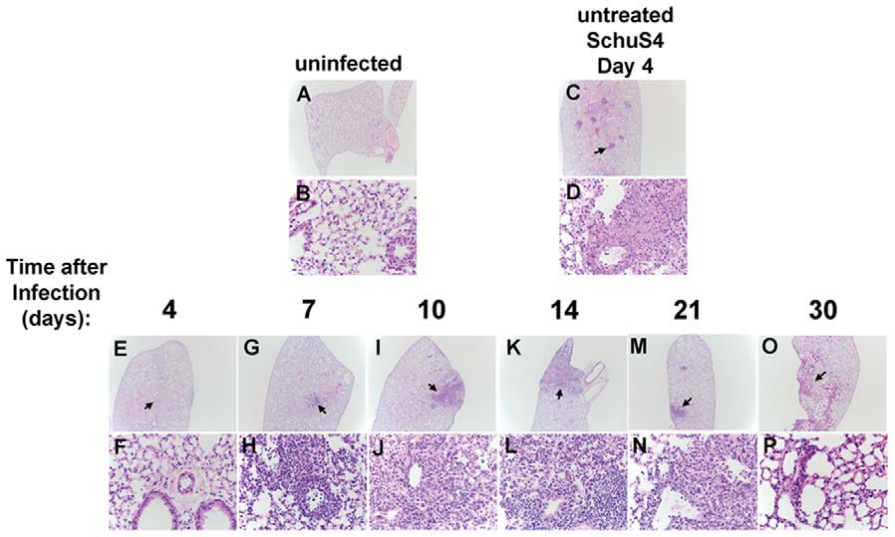
Pathological changes in the lungs of SchuS4 infected mice following antibiotic therapy. Mice were infected intranasally with 50 CFU SchuS4. Three days after infection mice were treated once daily with 5 mg/kg LVF diluted in 5% dextrose water intraperitoneally for 14 days. At the indicated time points, lungs from untreated, SchuS4 infected animals (C and D) and SchuS4 infected mice treated with LVF (E–P) were fixed, sectioned, strained with H&E and assessed for pathological changes. Uninfected mice (A and B) served as normal controls. Representative photomicrographs for each time point are shown. Plates A, C, E, G, I, K, M, and O are at 2× magnification and plates B, D, F, H, J, L, N, P are 40× magnification of the areas indicated by the arrows on the 2× plates.

**Figure 4:**
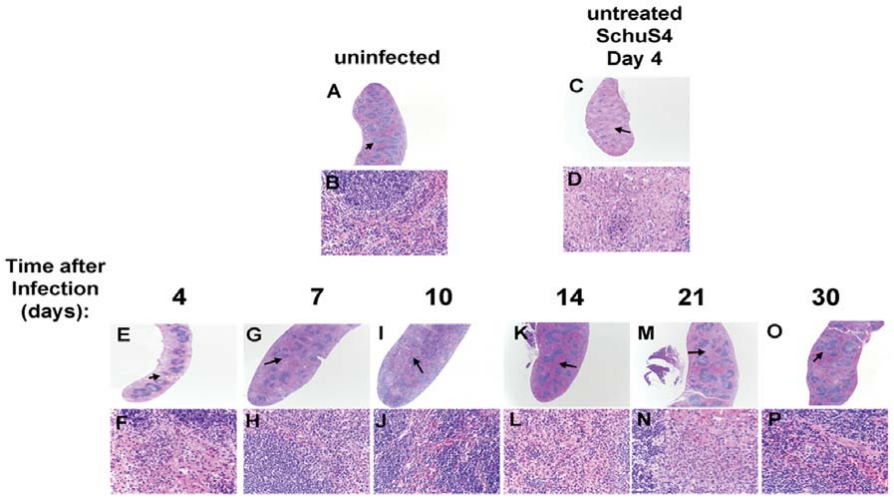
Pathological changes in the spleens of SchuS4 infected mice following antibiotic therapy. Mice were infected intranasally with 50 CFU SchuS4. Three days after infection mice were treated once daily with 5 mg/kg LVF diluted in 5% dextrose water intraperitoneally for 14 days. At the indicated time points, spleens from untreated, SchuS4 infected animals (C and D) and SchuS4 infected mice treated with LVF (E–P) were fixed, sectioned, strained with H&E and assessed for pathological changes. Uninfected mice (A and B) served as normal controls. Representative photomicrographs for each time point are shown. Plates A, C, E, G, I, K, M, and O are at 2× magnification and plates B, D, F, H, J, L, N, P are 40× magnification of the areas indicated by the arrows on the 2× plates.

We next assessed pathological changes in the lungs and spleens of SchuS4 infected mice following infection and treatment with LVF. Consistent with previous observations, by day 4 after infection, untreated animals have significant pathological changes in both the lung and spleen (Figure 3 and 4 [40]–[42]). In the lung, pulmonary lesions are consistent with necrotizing pneumonia with effacement of the pulmonary parenchyma and replacement with cellular and karyorrhectic debris, fibrin, and degenerate neutrophils (Figure 3C and D). Similarly, 4 days after SchuS4 infection untreated animals exhibit serious necrosis in the spleen with architecture of the spleen diffusely effaced and replaced by necrotic debris; there is also extensive loss of white pulp (Figure 4C and D). These changes are consistent with previous observations of pathological changes four days after Type A infection of Balb/c mice [43].

**Figure 5:**
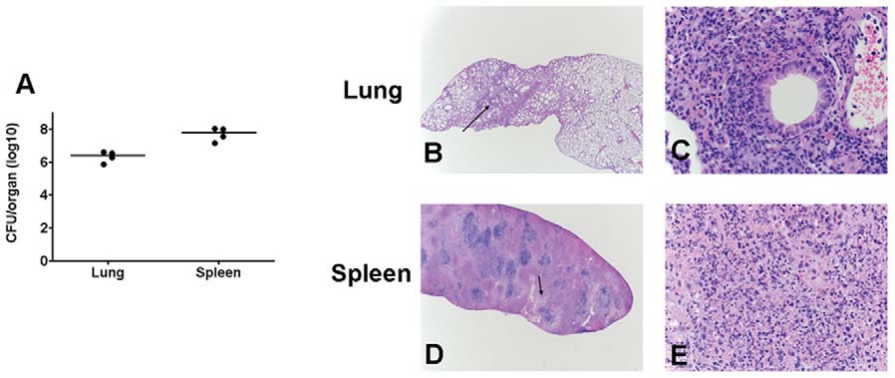
Bacterial loads and pathological changes in the lungs and spleens of SchuS4 infected mice following antibiotic therapy that succumb to infection. Mice were infected intranasally with 50 CFU SchuS4. Three days after infection mice were treated once daily with 5 mg/kg LVF diluted in 5% dextrose water intraperitoneally for 14 days. After antibiotic treatment, mice (n = 4) showing irreversible signs of illness were euthanized. Lungs and spleens were collected and assessed for bacterial loads (A) or pathological changes (B–E). Representative photomicrographs for each time point are shown. Plates B and D are at 2× magnification and plates C and E are 40× magnifications of the areas indicated by the arrow on the corresponding 2× plates.

In contrast to untreated animals, mice that received LVF have milder pathological changes at 4 days after infection. Specifically, pulmonary lesions in these animals consisted of multifocal neutrophilic bronchopneumonia. However, by day 7 after infection the nature of the lesions has changed. A multifocal subacute bronchopneumonia characterized by infiltrates of moderate numbers of macrophages, lymphocytes, and neutrophils that surround multiple bronchioles and expand the adjacent alveolar septae is evident in all lungs (Figure 3G and H). This non-progressing bronchopneumonia is also observed at 10 and 14 days after infection (Figure 3I–L). Twenty-one days after infection new changes in pulmonary pathology are observed compared to day 7–14, with a much larger influx of neutrophils admixed with smaller numbers of macrophages and lymphocytes (Figure 3 M and N). Many of the neutrophils are degenerate and there is cellular and karyorrhectic debris (necrosis) within the alveolar spaces. However, by day 30 after infection lesions have resolved and the lung tissue have returned to normal (Figure 3 O and P). In the spleens of SchuS4 infected mice treated with LVF, a similar pattern of slowly resolving pathology followed by recrudescences of lesions to those observed in the lungs of day 4 infected animals is apparent. Specifically, 4 days after infection, spleens of LVF treated mice have multifocal foci of viable and degenerate neutrophils within the red pulp and within many follicular centers (Figure 4E and F). There was also a mild depletion of lymphocytes within the white pulp. Seven days after infection the spleen is expanded by extramedullary hematopoiesis (EMH) that is composed predominantly of large numbers of blastic erythroid precursor cells and smaller numbers of nucleated red blood cells (Figure 4G and H). By day 10 after infection, the nature of the EMH has changed and is composed primarily of large numbers of nucleated red blood cells and fewer blastic cells in the red pulp (Figure 4 I and J). Within 14 days of infection the spleen has nearly returned to normal with some mild EMH (Figure K and L). However, at day 21 after infection there is a recrudescence of splenitis characterized by multifocal to coalescing foci of neutrophils and macrophages that efface the normal architecture (Figure 4M and N). In addition, there is lymphoid depletion that closely resembles that seen in the untreated mice at 4 days after infection. In mice that survive to 30 days after infection and cleared SchuS4 infection spleens are largely normal with only rare, small, lesions present (Figure 4O and P). In contrast to surviving mice, animals that succumb to infection between day 21 and 30 exhibited pathology in the lung and spleen indistinguishable from that observed in untreated animals 4 days after infection (Figure 5B–E).

### Survival of SchuS4 infection requires T cells and B cells

**Figure 6:**
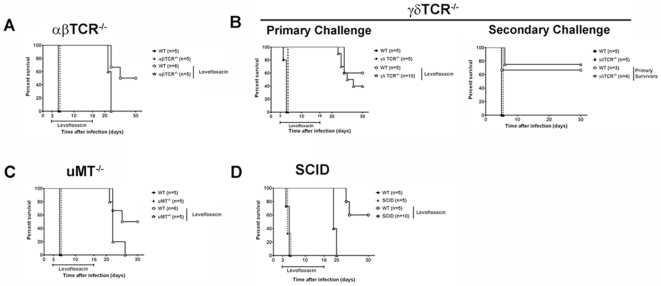
Cellular requirements for survival of tularemia. αβTCR^−/−^ (n = 5), γδTCR^−/−^ (n = 10), µMT^−/−^ (n = 5), SCID (n = 10) and C57Bl/6 (n = 5/experiment) (wild type, WT) mice were intranasally infected with 50 CFU/25 µl SchuS4. Three days after infection mice were treated once daily with 5 mg/kg LVF diluted in 5% dextrose water intraperitoneally for 14 days. * = p<0.05 compared to untreated mice and LVF treated αβTCR^−/−^, γδ TCR^−/−^, µMT^−/−^, and SCID mice. Data in each graph is representative of 2 experiments of similar design.

Previous studies have shown that both T cells and B cells participate in the control and resolution of infections mediated by attenuated strains of *F. tularensis* [34], [44]–[46]. The importance of these cellular subsets in control of primary infection with less virulent strains of *F. tularensis* has been further highlighted by the inability of severe combined immunodeficiency (SCID) mice (which do not have T or B cells, but possess NK cells as the primary effector cell population) to fail in their resolution of infection with attenuated *F. tularensis* [33]. Thus, we next determined if T and B cells played a similarly important role in survival of primary infection with virulent *F. tularensis*. Mice lacking αβTCR expressing T cells, e.g. CD4^+^ and CD8^+^ T cells, survive *F. tularensis* infection during administration of antibiotic. However, all αβTCR deficient animals succumb to infection within 4–5 days after cessation of LVF treatment (Figure 6A). Interestingly, 40% of mice that lack T cells bearing γδTCR survive primary SchuS4 infection (Figure 6B) and, of these survivors, 75% survive secondary infection with SchuS4 (Figure 6B).

We next examined what contribution conventional B cells make toward survival of SchuS4 infection. Similar to αβTCR^−/−^ animals, mice lacking mature, conventional B cells (µMT^−/−^) do not succumb to infection during treatment with LVF, but die after antibiotic therapy ended (Figure 6C). However, unlike mice lacking αβTCR, µMT^−/−^ do not require euthanasia until 9–10 days (as opposed to 4–5 days in αβTCR^−/−^ mice) after ending LVF treatment (Figure 6C).

Finally, using SCID mice we examined what effect absence of T and B cells would have on the course of SchuS4 infection. As observed in mice deficient for αβTCR, γδTCR, and µMT, SCID mice also survive infection during treatment with antibiotic. However, SCID mice succumb to infection within 4 days after administration of LVF ended (Figure 6D). Together these data suggest that neither T cells nor B cells are required to control *F. tularensis* infection during antibiotic therapy. However, both αβTCR^+^ T cells and conventional B cells are required to survive primary SchuS4 infection upon withdrawal of antibiotic. Finally, compared to αβTCR^+^ cells, γδTCR^+^ cells play a less pivotal role in survival of primary and sequential secondary infection.

### Sustained production of IL-12 in organs of SchuS4 infected mice

**Figure 7:**
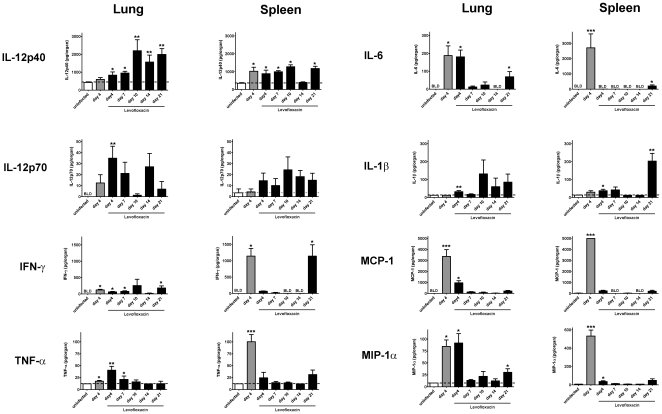
Cytokine production in target organs of SchuS4 infected mice following antibiotic therapy. C57Bl/6 mice were intranasally with 50 CFU SchuS4. Three days after infection mice were treated once daily with 5 mg/kg LVF diluted in 5% dextrose water intraperitoneally for 14 days. At the indicated time points animals (n = 5/group) were euthanized and lungs and spleens were homogenized. Homogenates were assessed for presence of the indicated cytokines using Flex Set Cytometric Bead Array assay. Uninfected mice served as negative controls. * = p<0.01 compared to uninfected mice. ** = p<0.01 compared to uninfected and infected, untreated animals. *** = p<0.05 compared to all other groups. Error bars represent SEM. Data is representative of 2 experiments of similar design.

Our data suggests that both T cells (αβTCR^+^ and γδTCR^+^) and B cells are required to survive primary infection with virulent SchuS4. Activation of T and B cells by cytokines can aid in generation of optimal protective responses. T cells and B cells can also secrete cytokines that contribute to killing microorganisms by activating host cells to initiate anti-microbial defenses. Therefore, we next assessed production and persistence of cytokines in the lungs and spleens of SchuS4 infected animals treated with LVF over time. At the indicated time points after infection, lung and spleen homogenates were assessed for cytokines using multiplex cytometric bead array and ELISA. IL-4, IL-5, and IL-17A were not detected in any sample at concentrations that were significantly different from uninfected controls at any time point after infection (data not shown). In agreement with previous reports, naïve, untreated, SchuS4 infected animals have significantly higher concentrations of TNF-α, IL-6, IL-1β, MCP-1(CCL2), and IL-10 in the lung and spleen on day 4 after infection compared to uninfected controls (Figure 7, data not shown and [47], [48]). Four days after infection, animals treated with LVF have similar concentrations of IL-6 and MIP-1α in their lungs compared to untreated SchuS4 infected animals (Figure 7). However, in contrast to untreated animals, spleens of mice treated with LVF have significantly less TNF-α, IL-6, IFN-γ, MCP-1, and MIP-1α 4 days after infection (Figure 7). As the course of disease progresses, IL-6, TNF-α, MCP-1, and MIP-1α are nearly undetectable in the lungs and spleens by day 7 after infection (Figure 7). In contrast, IL-12p40 was detected in these tissues at concentrations that are significantly higher than those observed in uninfected animals, even after 10 and 21 days of infection in the spleen and lung, respectively (Figure 7). Together these data suggest that extended SchuS4 infection correlates with cessation of production of selected pro-inflammatory cytokines and chemokines, e.g. TNF-α, IL-6, MCP-1 and MIP-1α and continued production of IL-12p40 in target tissues.

### IL-12 and IFN-γ are required to survive *F. tularensis* infection

**Figure 8:**
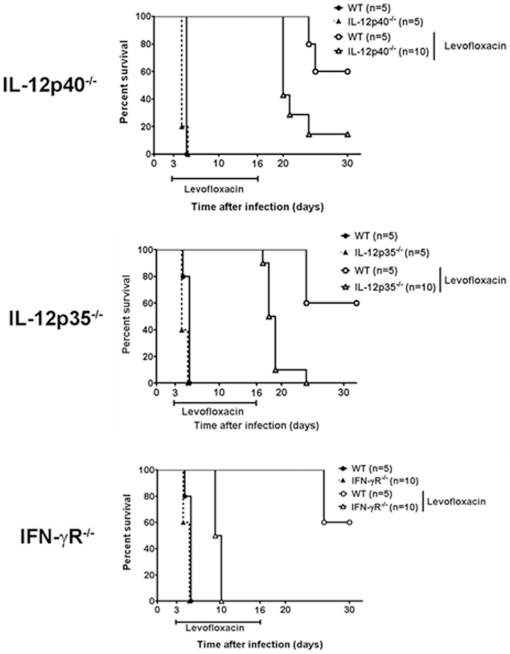
IL-12 and IFN-γ are required to survive tularemia. IL-12p40^−/−^ (n = 10), IL-12p35^−/−^ (n = 10), IFN-γR^−/−^ (n = 10) and C57Bl/6 (n = 5/experiment) (wild type, WT) mice were intranasally infected with 50 CFU/25 µl SchuS4. Beginning on day 3 after infection, mice were treated once daily with 5 mg/kg LVF diluted in 5% dextrose water for 14 days. * = p<0.05 compared to untreated mice and LVF treated IL-12p40^−/−^, IL-12p35^−/−^, IFN-γR^−/−^ animals. ** = p<0.05 compared to all other groups of mice. Data in each graph is representative of 2 experiments of similar design.

IL-12 can act on T and NK cells to produce IFN-γ [49] We and others have shown that IFN-γ can contribute to control of intracellular replication of virulent and attenuated strains of *F. tularensis* in vitro [50]–[52]. Furthermore, IFN-γ is required for survival of in vivo infection with attenuated strains of *F. tularensis* [22], [33]. The role of IL-12 in tularemia is unclear since one report suggests neither IL-12p35 nor IL-12p40 are required to survive infection with LVS, whereas others have shown both of these cytokines are essential [22], [23]. In our model, IL-12p40 is detected for up to 2 weeks in tissues of mice resolving SchuS4 infection (Figure 7). Thus, we hypothesized that IL-12 may play an important role in survival of infection with virulent *F. tularensis*. Similar to other immunodeficient mice, all untreated IL-12p40^−/−^ and IL-12p35^−/−^ that were not treated with LVF succumb to SchuS4 infection within 4–5 days of inoculation (Figure 8). The mean time to death for both groups of mice is not statistically different from untreated wild type animals. In the presence of LVF, SchuS4 infected IL-12p40^−/−^ and IL-12p35^−/−^ have significantly fewer survivors (10% and 0%, respectively) compared to wild type animals (Figure 8A and 8B), but do not succumb to disease until after LVF therapy ended. One IL-12p40^−/−^ animal remained after primary infection, thus we determined if this animal could survive a second SchuS4 challenge. The IL-12p40^−/−^ animal died on day 9 after secondary infection (data not shown). This suggests that IL-12p40 is also required to survive secondary SchuS4 infection.

As described above, IL-12 is tightly associated with production of IFN-γ. IFN-γ is well known for its role in protecting against intracellular pathogens (as reviewed, [53], [54]). Since IL-12 played a critical part in survival of SchuS4 infection we hypothesized that IFN-γ may also be required to survive infection with this bacterium. Interestingly, unlike all other genetically deficient mice tested in this study, animals lacking IFN-γR fail to survive SchuS4 infection even in the presence of antibiotic (Figure 8C). Thus, IL-12p40, IL-12p35, and IFN-γ are required to survive primary SchuS4 infection.

### Both CD4^+^ and CD8^+^ cells contribute to IFN-γ production during SchuS4 infection

**Figure 9:**
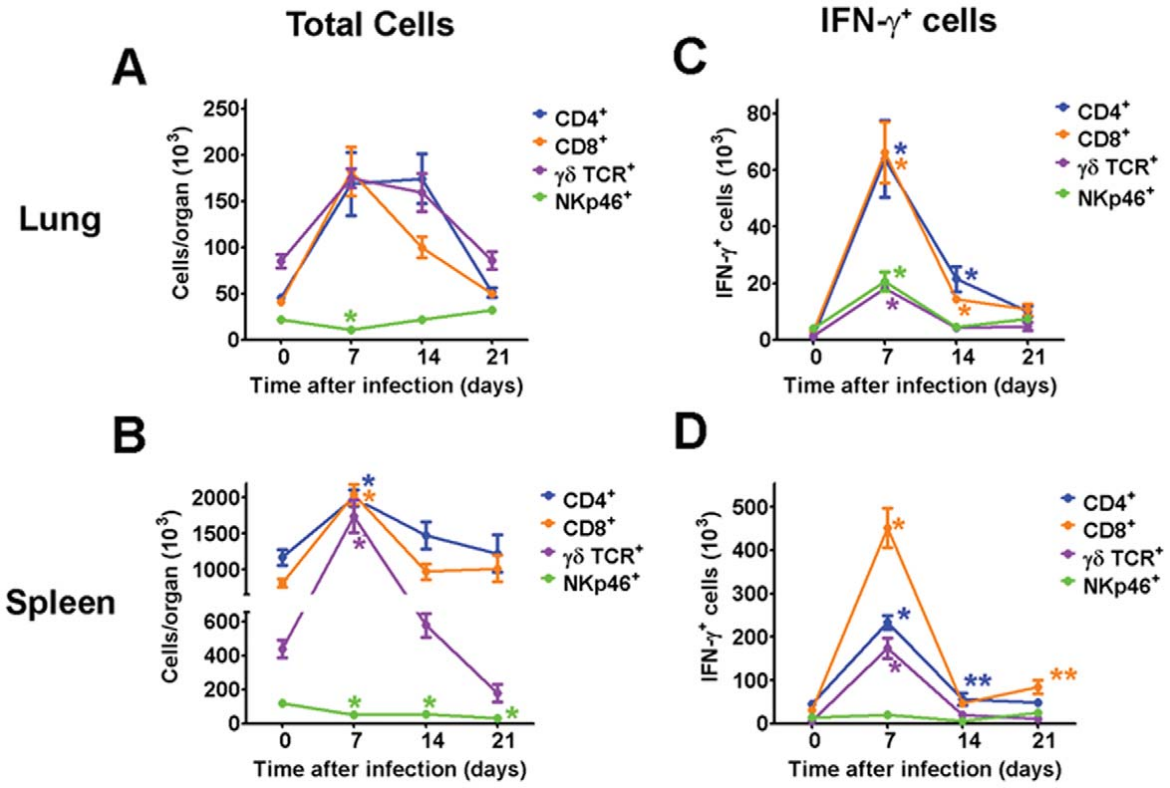
CD4^+^ and CD8^+^ cells are the primary producers of IFN-γ during SchuS4 infection. Mice were intranasally infected with 50 CFU/25 µl SchuS4. Beginning on day 3 after infection, mice were treated once daily with 5 mg/kg LVF diluted in 5% dextrose water for 14 days. At the indicated time points lungs and spleens were assessed for the indicated cells and IFN-γ by flow cytometry. * = p<0.05 compared to uninfected controls. The color of the asterisk correlates with the same color of data points depicted on the graph.

Data presented above indicates that both T cells and IFN-γ are required for survival of SchuS4 infection (Figure 6 and 8). Previous reports have shown that following infection with attenuated subspecies of *F. tularensis*, e.g. LVS, CD4^+^ and NK cells are primarily responsible for production of IFN-γ in the lung and spleen [55]–[58]. However, a similar analysis among naïve animals infected with Type A *F. tularensis* has not been reported. Therefore, we next determined which cell populations were responsible for producing IFN-γ during SchuS4 infection, if the cell type changed over time, and if there were differences in IFN-γ producing cells in the lung and spleen. We first assessed changes in the overall cell populations in the lung and spleen over time. In agreement with pathology reported above, there is a general expansion in the lymphocyte compartment in both the lung and spleen of SchuS4 infected animals within the first 7 days of infection (Figure 9A and B). However, this expansion did not include NK cells which were significantly reduced in both the lung and spleen at this time point compared to uninfected controls at day 7 and 14 after infection (Figure 9A and B). In the lung, there is evidence that the number of CD8^+^ cells are contracting by day 14 after infection and by day 21 CD4^+^, CD8^+^ and γδTCR^+^ lymphocytes had all returned to baseline numbers (Figure 9A). In the spleen, 14 days after infection the number of CD4^+^, CD8^+^ and γδTCR^+^ cells returned to numbers similar to uninfected controls (Figure 9B). The numbers of CD4+ and CD8+ cells did not significantly change after this time point. However, the number of splenic γδTCR^+^ continued to drop and was significantly lower at day 21 after infection compared to uninfected controls (Figure 9B). We then determined which populations of cells in the spleen and lung were producing IFN-γ. Within the first 7 days of infection we were able to detect significantly higher numbers of CD4^+^, CD8^+^ and γδTCR^+^ cell populations that were positive for IFN-γ in both the lung and spleen compared to uninfected controls (Figure 9C). However, more CD4^+^ and CD8^+^ are positive for IFN-γ than γδTCR^+^ cells in both the lung and spleen at this time point after infection (Figure 9). Additionally, although the overall numbers of pulmonary NK cells decrease 7 days after infection (Figure 9A) significantly more NK cells are positive for IFN-γ compared to uninfected controls at day 7 after infection (Figure 9C). In contrast, NK cells do not appear to make a contribution toward production of IFN-γ in the spleen at any of the time points we tested (Figure 9D). Although CD4^+^ and CD8^+^ cells dominate the IFN-γ response in both the lung and spleen after SchuS4 infection, we observed disparity in the contribution each of these cells made toward production of IFN-γ in each organ. For example, in the lung, we observe similar, significantly higher numbers of IFN-γ^+^CD4^+^ and IFN-γ^+^CD8^+^ cells at day 7 and 14 after infection compared to uninfected controls (Figure 9C). However, by day 21 after infection there are no differences in the number of IFN-γ^+^ cells in the lung compared to uninfected mice (Figure 9C). In contrast, at day 7 after infection we detect significantly more CD8^+^IFN-γ^+^ cells compared to all other cell types in the spleen (Figure 9). As the infection progresses, first splenic CD4^+^ cells are the predominate IFN-γ^+^ cells (day 14) only to be usurped by CD8^+^ cells at day 21 after infection (Figure 9D). We also assessed cellular populations in animals euthanized at day 25 after infection and found highly variable results with regard to both overall cell numbers and populations of cells producing IFN-γ. Specifically, when bacterial loads are low we observe similar numbers of CD4^+^ and CD8^+^ cells in the lung and spleen. Moreover, both CD4^+^ and CD8^+^ cells are positive for IFN-γ in the lung and spleen with slightly higher numbers of CD8^+^IFN-γ^+^ cells in the spleen (Figure S2). Animals that do not control bacterial replication had an overall increase in CD4^+^ and CD8^+^ cells that was similar to the response observed at day 7 after infection (Figure S2). The IFN-γ response in the lungs of these animals is dominated by CD4^+^ cells with only a modest contribution made by the CD8^+^ cell population (Figure S2). In the spleen, all cell types assessed are positive for IFN-γ among animals with high bacterial loads with no clear preference toward cell type responding to infection (Figure S2). Thus, at day 25 after infection there are some animals that are clearly capable of controlling the recrudescence of SchuS4 growth following removal of antibiotic whereas other animals cannot. However, the failure of some animals to control SchuS4 replication is not due to the inability of host cells to respond to infection and produce IFN-γ, but may lie in the proportion of the cellular response or another undefined mechanism. Finally, our data suggests that CD4^+^ and CD8^+^ cells make equal and transient contributions toward production of IFN-γ in the lung during the first two weeks of infection, but are not sustained. In the spleen, CD4^+^ and CD8^+^ cells vary in their contribution toward production of IFN-γ over the course of infection with CD8^+^IFN-γ^+^ cells persisting at later time points after infection.

## Discussion

In the current report, we utilized antibiotic therapy to extend the course of infection with virulent *F. tularensis* ssp. *tularensis* strain SchuS4 in order to identify components of the immune response that are required for survival of primary infection with SchuS4. Using this approach, we successfully developed a model in which, upon withdrawal of the antibiotic, the host response was ultimately responsible for clearance of the pathogen. This feature was evident in the uniform rise of bacterial numbers upon cessation of antibiotic therapy in all animals. This was followed by complete clearance of the bacterium and survival in 60% of the animals. We then employed this in vivo model to identify host factors required for survival of virulent *F. tularensis* infection. Consistent with previous observations using mice with different immunodeficiencies than those examined here, we observe that untreated, immunodeficient, mice infected with SchuS4 do not display rates of survival or mean time to death that are significantly different from untreated wild type animals (Figure 6 and 8, [40]–[42]. However, treatment with antibiotic extends mean time to death and enhances survival of SchuS4 infected animals, thus allowing better assessment of the role of specific host cells and molecules. This model provides several novel findings which include a requirement for conventional B cells, and to a lesser extent, γδTCR^+^ cells for survival of primary infection with virulent *F. tularensis*. Further, our results clarify the role for IL-12p40 and IL-12p35 following infection with *F. tularensis* by demonstrating that both of these cytokines are required to survive primary SchuS4 infection. Finally, in agreement with previously published findings using attenuated strains of *F. tularensis*, αβTCR^+^ cells and IFN-γ are required to survive infection ([32], [33] and Figure 6 and 8).

Our most striking finding was the requirement for IFN-γ. Unlike all other knockout mice tested, mice deficient for IFN-γ receptor are unable to survive *F. tularensis* infection during antibiotic therapy. It has been established that IFN-γ is important for resolution of a wide range of infections caused by intracellular pathogens. For example, exogenous administration of recombinant IFN-γ contributes to the control of infection caused by *Listeria monocytogenes* and *Mycobacterium tuberculosis* [59], [60]. Alternatively, neutralization of this cytokine increases bacterial loads and reduces survival in mice infected with either of these bacteria [61], [62]. IFN-γ has also been found to play a key role in control of infections mediated by attenuated strains of *F. tularensis* [33], [50], [63]. The mechanism of action for IFN-γ is to activate professional antigen presenting cells, such as macrophages, to kill phagocytosed microorganisms. With respect to infection with *F. tularensis*, we and others have shown that one mechanism by which IFN-γ mediates killing of this intracellular pathogen is by activating pathways that generate both reactive nitrogen species (RNS) and reactive oxygen species (ROS) [51], [52]. However, following in vitro infection of macrophages with virulent *F. tularensis*, IFN-γ must be present either before cells are infected or within several hours after infection to contribute to control of intracellular replication of *F. tularensis* [51], [52], [64]. Similarly, results from our in vivo model presented herein suggest that IFN-γ must be present at early time points after in vivo infection to ensure survival of tularemia.

Given the importance of IFN-γ in survival of virulent *F. tularensis* infection, identification of the cellular source of this cytokine, as well as soluble mediators that contribute toward production of IFN-γ, is critical toward understanding the requirements of protection against tularemia. IFN-γ can be made by a variety of cell types including, γδTCR^+^, αβTCR^+^, and NK cells. In the current manuscript, we provide direct evidence that αβTCR^+^ cells and, to a smaller extent, γδTCR^+^ cells are necessary to survive primary SchuS4 infection (Figure 3). Thus, either of these cell types may serve as a cellular source for IFN-γ. However, since 0% of mice lacking αβTCR^+^ cells survived primary SchuS4 infection compared to survival of 30% of γδTCR^−/−^ mice, our data suggests that the role for αβTCR^+^ cells appears to be more critical than the presence of γδTCR^+^ cells. In agreement with this hypothesis, higher numbers of CD4^+^ and CD8^+^ cells were positive for IFN-γ throughout SchuS4 infection in both the lung and spleen compared to γδTCR^+^ cells. This does not completely preclude a role for γδTCR^+^ cells in defense against *F. tularensis*. For example, although γδTCR^+^ cells are not required to survive infection with attenuated *F. tularensi*s, there is evidence that these cells contribute to control of bacterial replication in the lung [65]. In that report, the proposed mechanism of action of γδ T cells against *F. tularensis* infection was secretion of IL-17A and not direct secretion of IFN-γ. IL-17A acts to support Th1 type immune responses, induce production of anti-microbial peptides, and enhance neutrophil expansion (as reviewed, [66]). We failed to observe production of IL-17A in lung or spleen homogenates that was significantly different from uninfected controls at any time point after infection (data not shown). However, we did not directly measure IL-17A production on a cellular level. This more sensitive method of detecting IL-17A may reveal a similar, small role and function for γδTCR^+^ cells during infection with virulent *F. tularensis* as that observed with attenuated bacteria.

Among αβTCR^+^ cells, i.e. invariant NKT (iNKT) cells, CD4^+^ T cells, and CD8^+^ T cells, all can produce IFN-γ [67]–[70]. The role of iNKT cells in tularemia has not been defined. However, iNKT cells have been shown to play a role in mediating protection against other pulmonary pathogens following secretion of IFN-γ [71]–[73]. Thus, it is reasonable to speculate that these cells may also contribute to control of virulent *F. tularensis*.

Presence of αβTCR^+^, CD4^+^, and CD8^+^ T cells have been shown to be important for control of infection with attenuated *F. tularensis*. Additionally, the presence of these cells have been show to aid in mediating protective immunity against virulent *F. tularensis* in vaccinated animals [32], [34], [46]. However, work examining the cellular source of IFN-γ following infection with LVS has suggested that CD4^+^ cells are the cell type primarily responsible for producing this cytokine at later time points in infection [55], [56], [58]. In the current study, our data suggests that control of SchuS4 replication in the spleen following delivery of levofloxacin is correlated with the presence of CD8^+^ IFN-γ^+^ and CD4^+^ IFN-γ^+^ cells in this tissue. This result is consistent with our previous observation in which expansion of IFN-γ producing CD4^+^ and CD8^+^ T cells in the spleens of LVS vaccinated animals following infection with SchuS4 correlated with survival [74]. Together these data suggest that SchuS4 provokes a more complex cellular response than that observed with attenuated subspecies of *F. tularensis*. Understanding the relative contribution of both CD4^+^ and CD8^+^ T cell responses, rather than the primary CD4^+^ response observed in LVS infected animals, is critical for development of novel vaccines and the convalescent model described herein might aid in that process. Identification of the specific contribution of CD4^+^ T cells, CD8^+^ T cells and iNKT cells toward control of *F. tularensis* infection is currently under intense study in our laboratory.

In addition to determining the cellular source of IFN-γ, it is also important to identify the soluble mediators that contribute to production of this cytokine. There are several cytokines that can promote production of IFN-γ from αβTCR^+^ cells including IL-12p70 and IL-18. Among these, IL-12p70 represents one of the most well studied cytokines. IL-12p70 is a heterodimer made up of IL-12p40 and IL-12p35. IL-12p35 is constitutively expressed whereas IL-12p40 requires activation of the cell prior to its production. In addition to their function as IL-12p70, each of these proteins can act independently or pair with other binding partners to form cytokines with distinct functions. For example, in the mouse IL-12p40 can be present as monomers or homodimers that act as antagonists for IL-12p70 due to its high affinity for the IL-12R [75]. IL-12p40 can also form heterodimers with p19 to form IL-23. IL-23 has emerged as an important cytokine for the production of IL-17 and the subsequent generation of Th17 type immune responses dominated by inflammation and granulocytic infiltrate [76].

Previously, it was shown that neither IL-12p35 nor IL-12p40 was required to survive infection with attenuated *F. tularensis*. This suggested that IL-12p35 and, by extension, IL-12p70 was not required for survival of attenuated *F. tularensis* infection. In contrast, a more recent report showed that both IL-12p35 and IL-12p40 were required for survival of LVS infection [23]. Although the differing routes of infection used in these studies may have contributed to the dramatically different outcomes observed by each lab, the fact remains that it is unclear what role, if any, IL-12 played in infection with virulent *F. tularensis*. Results presented herein agree with the more recent report in that both IL-12p35 and IL-12p40, and thus IL-12p70, are required for survival of tularemia. However, since both IL-12p40 and IL-12p35 can act independently of each other to mediate immune responses, it is possible that the role of these proteins may be distinct from their function in forming IL-12p70.

For example, it is possible that IL-12p35 may be working independently to promote early inflammation, as recently described in herpes stromal keratitis [77]. Alternatively, IL-12p35 may partner with EBI3 to form IL-27. IL-27 is provoked by a number of intracellular pathogens and has both pro- and anti-inflammatory functions that contribute to control of infection (as reviewed, [78]). The specific function of IL-12p35, IL-12p40, and IL-12p70 in tularemia are currently under investigation in our laboratory.

Finally, we determined that conventional B cells are required for survival of SchuS4 infection (Figure 6). One function of B cells is the production of antibody to aid in control of infection. Presence of antibodies has been proposed to be important for protection against tularemia. Indeed, passive transfer of immune serum and monoclonal antibodies has successfully protected mice against challenge with attenuated LVS [79]–[82]. In rats, passive transfer of immune serum successfully protected against SchuS4 infection [83]. Early studies in humans found that passive transfer of hyperimmune serum generated in horses aided in control of active cases of tularemia [38]. Thus, antibody can clearly support control and clearance of *F. tularensis*. However, during infection of naïve animals with virulent *F. tularensis* the requirement for antibody versus other, unrelated activity exerted by B cells has not been defined. B cells have additional functions outside of antibody production that include production of cytokines such as IL-6, antigen presentation, and regulation of dendritic cells (as reviewed, [84]). Thus, B cells may serve in other ways to mediate protection against *F. tularensis* infection that are unrelated to antibody production. In support of this hypothesis, we previously demonstrated that B cells, but not antibody, were critical for survival of LVS infection in mice [44], [45]. Importantly, recent preliminary data generated in our laboratory using animals with specific defects in their ability to produce and secrete antibody suggests antibody is not required for survival of primary SchuS4 infection (CM Bosio, unpublished observations). Thus, while antibody can contribute to the clearance of *F. tularensis*, presence of this host component may not be necessary to successfully resolve infection. Rather, B cells may have other undefined functions to contribute to survival of tularemia.

Over the past 10 years interest in disease caused by virulent *F. tularensis* has increased, in part due to its potential as a biological weapon [85]. Given the similarities of infectious dose required to cause disease and the development of similar pathology between human and mice exposed to virulent *F. tularensis* in the pulmonary compartment, the mouse model of tularemia is a valuable tool for the study of this disease in vivo. However, comprehensive investigation of the cellular and soluble requirements for survival of virulent *F. tularensis* infections were not possible due to the rapid mean time to death following exposure of the animals to very low doses of bacteria. Data presented herein demonstrates that low dose antibiotic therapy can be used as a method to overcome this hurdle. Thus, we can now gain a better understanding of requirements for protection and survival of primary tularemia. Furthermore, our model is also reminiscent of observations made in humans following use of antibiotics for treatment of tularemia, where both relapse and modest, but incomplete, protection against re-infection have been noted [86]–[89]. Thus, our model will provide a unique opportunity to study infection with virulent *F. tularensis* that will reveal novel and unexpected functions for host factors involved in both the resolution and exacerbation of tularemia.

## Materials and Methods

### Ethics Statement

This study was carried out with strict accordance with the recommendations made by the National Institutes of Health. The protocol was approved by the animal care and use committee at the Rocky Mountain Laboratories/NIAID/NIH, protocol number 2009-66.

### Mice

Specific-pathogen-free, 6–8 week old C57Bl/6J (wild type), B6.129S2-*Tcra^tm1Mom^*/J (αβTCR^−/−^), B6.129P2-*Tcrd^tm1Mom^*/J (γδTCR^−/−^), B6.129S2-*Ighm^tm1Cgn^*/J (µMT^−/−^), B6.CB17-*Prkdc^scid^*/SzJ (SCID), B6.129S1-Il12b^<tm1Jm>^/J (IL-12p35^−/−^) and B6.129S1-*Il12a^tm1Jm^*/J (IL-12p40^−/−^)(n = 5–10/group) were purchased from Jackson Laboratories (Bar Harbor, ME). Mice lacking IFN-gamma receptor (IFN-γR^−/−^) were bred at the Rocky Mountain Laboratories (RML). Mice were housed in sterile microisolater cages in the BSL-3 facility at the RML. All mice were provided sterile water and food *ad libitum* and all research involving animals was conducted in accordance with Animal Care and Use guidelines and animal protocols were approved by the Animal Care and Use Committee at RML. Following infection mice were regularly monitored and euthanized at the first signs of illness in accordance with Animal Care and Use guidelines and animal protocols approved by the Animal Care and Use Committee at RML.

### Bacteria

*Francisella tularensis* ssp. *tularensis* strain SchuS4 was kindly provided by Jeannine Peterson, Ph.D. (Centers for Disease Control, Fort Collins, Colorado). SchuS4 was cultured in modified Mueller-Hinton broth (Mueller-Hinton broth supplemented with CaCl_2_, MgCl_2_, 0.1% glucose, 0.025% ferric pyrophosphate and 2% Medium Enrichment [50% glucose, 167 mM L-cysteine-HCl, 68 mM L-glutamine, mM adenine, 376 µM nicotinamide adenine dinucleotide, 7 µM Vitamin B_12_, 217 µM thiamine pyrophosphate, 160 µM guanine-HCl, 50 µM ferric nitrate, 95 µM aminobenzoic acid, 9 µM thiamine hydrocholride]) at 37°C with constant shaking overnight, aliquoted into 1 ml samples, frozen at −80°C and thawed just prior to use as previously described [9]. Frozen stocks were titered by enumerating viable bacteria from serial dilutions plated on modified Mueller-Hinton (MMH) agar as previously described [33], [44]. The number of viable bacteria in frozen stock vials varied less than 1% over a 12 month period.

### Infection of mice and administration of antibiotic

Mice were infected intranasally (i.n.) with approximately 50 CFU/25 µl *F. tularensis* SchuS4 as previously described [13]. Briefly, bacteria were thawed and diluted in PBS. Mice were anesthetized by intraperitoneal injection of 100 µl of 12.5 mg/ml ketamine+3.8 mg/ml xylazine. Approximately 50 CFU was administered into the nares of each mouse in a total volume of 25 µl. Actual inoculum concentration was confirmed by plating a portion of the inoculum onto MMH agar plates, incubating plates at 37°C with 5%CO_2_ and enumerating colonies. At the indicated time points after infection mice were treated with either 40 or 5 mg/kg levofloxacin (LVF) (Ortho-McNeil Pharmaceutical, Raritan, NJ). Levofloxacin was prepared by diluting stock antibiotic to the appropriate concentration in 5% dextrose water immediately prior to use. Mice were injected intraperitoneally with 100 µl of diluted LVF once daily for 14 days. As indicated, mice that survived primary SchuS4 challenge were given a second intranasal infection of *F. tularensis* SchuS4 (50 CFU/25 µl) 30 days after the initial infection. All mice were monitored for 30 days after each infection. Following infection mice were monitored regularly and euthanized at the first signs of illness. All experiments using animals were performed in accordance with protocols approved by the Animal Care and Use Committee at RML.

### Collection of tissue homogenate and enumeration of bacteria

Bacteria were enumerated from the lungs and spleens as previously described [9], [13]. Briefly, organs were aseptically collected and placed in ice cold homogenization buffer (150 mM Tris-HCl, 5 mM EDTA, 10 mM Trizma-base) supplemented with a 1:100 dilution of Phosphatase Inhibitor cocktail I, Phosphatase Inhibitor cocktail II and Protease Inhibitor cocktail III (all from AG Scientific, San Diego, CA). Organs were immediately homogenized by grinding tissues through a sterile S/S Type 304 #60 wire mesh screen (Billeville Wire Cloth Co., Cedar Grove, New Jersey) using a 3 ml syringe plunger. A portion of the resulting homogenate was immediately serially diluted in PBS and plated on MMH agar for enumeration of bacterial loads. The remaining homogenate was centrifuged at 14,000×g for 30 min at 4°C. The resulting supernatants were sterile filtered through 0.2 µm syringe filters (Millipore Ireland LTD, Cork, Ireland) and stored at −80°C before assessment for cytokines.

### Assessment of pathological changes in tissues

Tissues were fixed in 10% neutral buffered formalin for a minimum of 24 hours. Tissues were embedded in paraffin, placed in cassettes, and processed with a Sakura VIP-5 Tissue Tek, on a 12 hour automated schedule, using a graded series of ethanol, xylene, and ParaPlast Extra. Embedded tissues are sectioned at 5 µm and dried overnight at 42°C prior to staining. Tissues sections were stained with hematoxylin and eosin (H&E) and examined on an Olympus BX51 light-microscope equipped with a Olympus DP722 camera and associated cellSens Dimension 1.4.1 software. Tissues were assessed by a board certified pathologist. Extent of necrosis, neutrophilia, lymphocyte depletion, extramedullary hematopoiesis, and distribution of lesions was scored on a scale of 0–5.

### Isolation of lung and spleen cells

Lung cells and splenocytes were collected as previously described with the following modifications [74]. Lung cells and splenocytes were resuspended in FACS buffer prior to flow cytometric analysis or DMEM supplemented with 10% heat-inactivated fetal calf serum (FCS), 0.2 mM L-glutamine, 1 mM HEPES buffer and 0.1 mM nonessential amino acids (all from Invitrogen, Carlsbad, CA) (cDMEM) prior to addition to tissue cultures. Total live cells from the lungs and spleens were enumerated using trypan blue and a TC10 Automated Cell Counter (Bio-Rad Laboratories, Hercules, CA).

### Flow Cytometry

Lung and splenocyte populations were assessed by flow cytometry as previously described [74]. Briefly, the following antibodies in various combinations were used for flow cytometric analysis: APC CD4, PerCPCy5.5 CD8, FITC NKp46, PeCy7 B220 and APC γδTCR (all from BD Biosciences, San Jose, CA). Staining was performed in FACS buffer at room temperature. Following staining, cells were washed and fixed in 1% paraformaldehyde for 30 minutes at 4°C. Cells were washed a final time, resuspended in FACS buffer and stored at 4°C until analyzed. Samples were collected using a LSRII flow cytometer (BD Biosciences). Analysis gates were set on viable unstained cells and were designed to include all viable cell populations. Approximately 10,000 gated events were analyzed for each sample. Isotype control antibodies were included when analyses and panels were first being performed to assure specificity of staining, but were not routinely included with each experiment. Data was analyzed using FlowJo software (Treestar, Ashland, OR).

Intracellular cytokines were detected by flow cytometry as previously described [14]. Single cell suspensions of lungs and spleens were resuspended in cDMEM at 2×10^5^ per well in 96-well plates in the presence of 10 µg/ml Brefeldin A, 10 ng/ml phorbol 12-myristate 13-acetate and 1 µg/ml ionomycin (all from Sigma) at 37°C/5% CO_2_ for 4 hours. Following incubation, cells were washed once and resuspended in FACS buffer and stained for CD4, CD8, γδTCR and NKp46 as described above. Then cells were fixed in 2% paraformaldehyde in PBS for 10 min at 37°C/7% CO_2_ and washed twice more in “perm buffer” (FACS buffer supplemented with 0.25% saponin [Sigma-Aldrich]). Cells were incubated for 20 min at room temperature with anti-mouse IFN-γ (PE; clone XMG1.2) or PE conjugated rat IgG (isotype control) (both from BDBiosciences). Cells were washed twice in perm buffer, fixed in 1% paraformaldehyde for 30 min, and then resuspended in FACS buffer and stored at 4°C until analysis. Cells were acquired and analyzed using a LSR II flow cytometer (BD Biosciences) and FlowJo Software (Treestar).

### Measurement of cytokines

Organ homogenates were assayed for the presence of TNF-α, IL-6, IL-10, IL-1β, IL-12p70, IL-17A, IL-5, IFN-γ, MCP-1, and MIP-1α, by Cytometric Bead Array using a LSRII multiparameter flow cytometer and FCAP Array Software (all from BD Biosciences) according to the manufacturer's instructions. IL-4 and IL-12p40 was assessed by commercially available ELISA according to manufacturer's instructions (R&D Systems).

### Detection of SchuS4 serum antibodies

Serum was collected from mice that survived SchuS4 infection and LVF treatment 30 days after challenge. Presence of serum IgG antibodies that recognize antigens in whole cell lysate prepared from SchuS4 were detected as previously described [74].

### Statistical Analysis

Statistical differences between two groups were determined using an unpaired t test with the significance set at p<0.05. For comparison between three or more groups, analysis was done by one-way ANOVA followed by Tukey's multiple comparisons test with significance determined at p<0.05. Significance in survival between groups was determined using Log-Rank (Mantel-Cox) test with significance determined at p<0.05.

## Supporting Information

Figure S1 **Convalescent mice develop anti-SchuS4 antibodies.** Thirty days after challenge surviving mice were euthanized and blood was collected via cardiac puncture. Serum was isolated and tested for IgG antibodies directed against SchuS4 whole cell lysate by ELISA. Uninfected mice served as negative control. Data is representative of 2 experiments of similar design.
(TIF)

Figure S2 **Variable bacterial loads and cellular responses in mice recovering from SchuS4 infection in the absence of antibiotics.** Mice were intranasally infected with 50 CFU/25 µl SchuS4. Beginning on day 3 after infection, mice were treated once daily with 5 mg/kg LVF diluted in 5% dextrose water for 14 days. Twenty-five days after infection lungs and spleens were assessed bacterial loads (A) for the indicated cells and IFN-γ by flow cytometry (B and C). Bacterial loads and cellular changes for individual mice are depicted on each graph.
(TIF)
