# Supplementary figures and images for: Generation of a Convalescent Model of Virulent Francisella tularensis Infection for Assessment of Host Requirements for Survival of Tularemia
Source: PLoS One. 2012 Mar 12;7(3):e33349. doi: 10.1371/journal.pone.0033349 (PMC3299770; doi:10.1371/journal.pone.0033349)

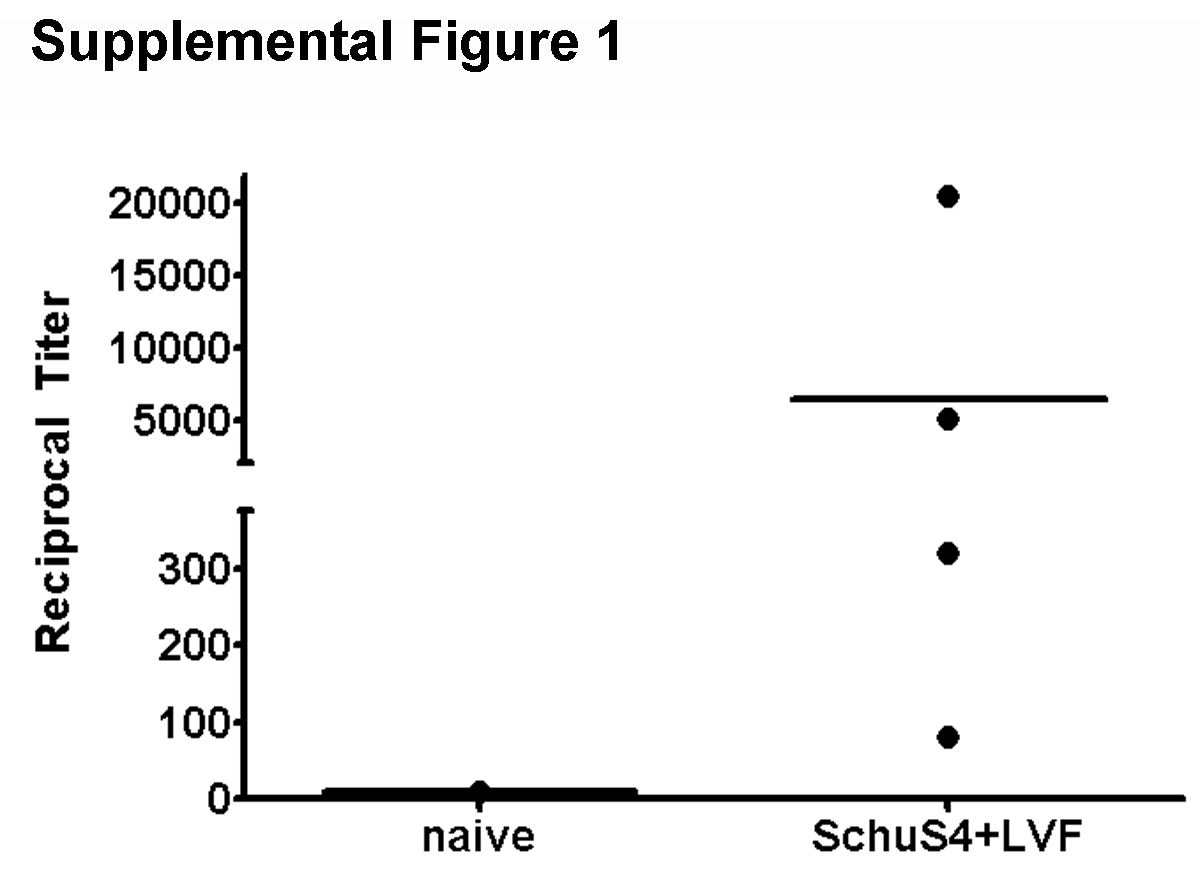

Supplement: Figure S1 — Convalescent mice develop anti-SchuS4 antibodies. Thirty days after challenge surviving mice were euthanized and blood was collected via cardiac puncture. Serum was isolated and tested for IgG antibodies directed against SchuS4 whole cell lysate by ELISA. Uninfected mice served as negative control. Data is representative of 2 experiments of similar design. (TIF) [file pone.0033349.s001.tif]

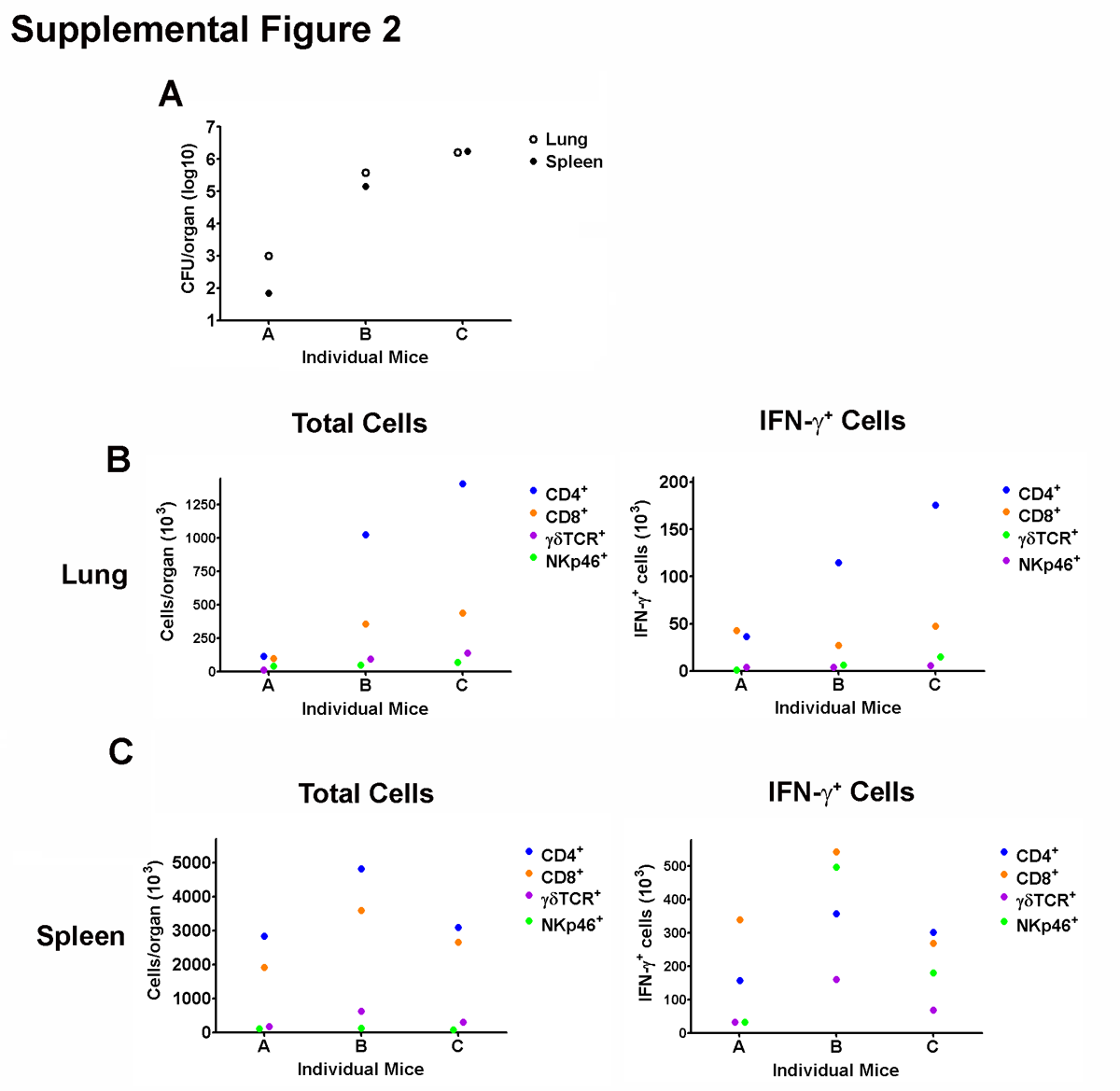

Supplement: Figure S2 — Variable bacterial loads and cellular responses in mice recovering from SchuS4 infection in the absence of antibiotics. Mice were intranasally infected with 50 CFU/25 µl SchuS4. Beginning on day 3 after infection, mice were treated once daily with 5 mg/kg LVF diluted in 5% dextrose water for 14 days. Twenty-five days after infection lungs and spleens were assessed bacterial loads (A) for the indicated cells and IFN-γ by flow cytometry (B and C). Bacterial loads and cellular changes for individual mice are depicted on each graph. (TIF) [file pone.0033349.s002.tif]
